# *Cutibacterium acnes* in confirmed and probable deep shoulder infections

**DOI:** 10.1177/17585732251359946

**Published:** 2025-07-17

**Authors:** Per Malmberg, Lena Serrander, Vendela M Scheer, Jens Nestorson, Johan H Scheer

**Affiliations:** 1 Department of Orthopaedic Surgery in Västervik, Kalmar County Council, 4566 Linköping University, Linköping, Sweden; 2 Department of Biomedical and Clinical Sciences, 4566 Linköping University, Linköping, Sweden; 3 Department of Medical Microbiology in Linköping, 4566 Linköping University, Linköping, Sweden; 4 Department of Anaesthesia, Perioperative and Intensive Care in Linköping, 4566 Linköping University, Linköping, Sweden; 5 Department of Orthopaedic Surgery in Linköping, 4566 Linköping University, Linköping, Sweden

**Keywords:** *Cutibacterium acnes*, *C acnes*, shoulder surgery, postoperative shoulder infection, surgical site infection

## Abstract

**Introduction:**

*Cutibacterium acnes* is frequently detected in tissue cultures of the shoulder, often in the absence of clear signs of infection. The aim of this retrospective study was to investigate the extent to which this bacterium appeared as the sole identifiable source of confirmed deep postoperative infections.

**Methods:**

From a database containing all positive deep tissue cultures from the shoulder region at our department between 2015 and 2021. In total, 106 cases in which ≥2 positive cultures of the same bacterium were identified. Medical records were subsequently reviewed for evidence of a confirmed infection, defined as having ≥2 positive cultures of the same species alongside either a draining sinus or pus observed intraoperatively.

**Results:**

*C acnes* was commonly detected in 63% of cases. Polymicrobial growth was identified in 21/106 cases. In confirmed infections, *C acnes* was the sole identifiable pathogen in 32% of cases. Beyond 3 months postoperatively both monomicrobial cases of *C acnes* and *Staphylococcus aureus* were identified in confirmed infections. Men were more likely to exhibit *C acnes* growth (*p* = 0.012).

**Conclusion:**

*C acnes* is a clinically relevant pathogen, albeit slow growing, and is capable of independently causing significant deep surgical site infections.

## Introduction

*Cutibacterium acnes* is the most commonly cultured bacterium following shoulder surgery.^1^ However, opinions on its role vary – ranging from being a benign commensal,^2^ possibly lying dormant within the joints or tissues prior to surgery,^3,4^ to a pathogen capable of independently causing deep surgical site infections (SSIs) that necessitate secondary surgical intervention and antibiotic treatment. A key source of this controversy stems from the diverse clinical manifestations associated with its presence. In prosthesis revision surgery due to pain, implant loosening or stiffness, 25–50% of shoulders are likely to yield positive cultures.^5–11^ Notably, its colonisation is frequently associated with the absence of clinical, laboratory or radiological indicators of infection. Given that SSIs are frequently polymicrobial, some suggest that *C acnes* may merely be an ‘innocent bystander’,^5^ a contaminant in the culturing process^12^ rather than a cause of harmful infections.^2^

The diagnosis of a deep implant-related SSI is often vaguely defined in different, often retrospective, studies. In recent years, consensus guidelines for prosthetic joint infection and fracture-related infection (FRI) have been largely agreed upon by professional societies comprising of orthopaedic surgeons, infectious disease specialists and microbiologists.^13–15^ In these guidelines infections are classified as ‘confirmed’ or ‘suggestive/likely’. However, throughout these guidelines, two or more positive cultures for the same organism is equated with an infection, regardless of whether the shoulder is merely colonised – as in incidental findings – or truly infected.

The aim of this study was to investigate the bacterial flora of shoulder infections in a retrospective cohort. For the purposes of this study, a confirmed infection is being defined as two or more positive cultures of the same bacterium, in conjunction with at least one additional confirmatory criterion listed in Table 1, in order to distinguish these from cases where there was uncertain clinical suspicion of an active infectious process.

**Table 1.** Adapted criteria for deep SSI.^10–12^

Confirmatory criteria	Suggestive criteria
Wound breakdown^a^	Elevated lab (CRP, Leukocyte count, ESR)
Fistula/sinus	Radiologic signs of infection
Pus during surgery	Clinical signs of infection (redness, swelling, pain)
Two or more cultures positive for the same organism	

SSI: surgical site infection; CRP: C-reactive protein; ESR: erythrocyte sedimentation rate

^a^ Direct communication between the external environment and the bone and/or implant.

## Materials and methods

This is a retrospective study of patients having secondary surgery of the shoulder following a prior arthroplasty, fracture fixation, or soft tissue surgery – such as open rotator cuff repair or acromioclavicular (AC) ligament reconstruction. The inclusion criterion was the presence of two or more positive deep tissue cultures obtained from the shoulder region. The Department of Clinical Microbiology maintains a database of all patients with at least one positive deep tissue culture, along with the anatomical region from which the samples were obtained. All patients marked as having ‘shoulder’ samples in this database between 2015 and 2021 were identified. Five deep tissue samples were collected during any revision surgery. Consequently, the cohort comprises cases that underwent surgery for the following indications:

1. Revision of a shoulder arthroplasty for any reason, for example loosening, instability etc.
2. Removal of hardware due to pain or discomfort following fracture or reconstructive surgery.
3. Secondary surgical intervention due to non-union, malunion, failed fracture fixation or failed ligament repair/reconstruction.
4. Debridement, antibiotics and implant retention procedures performed in response to varying degrees of suspicion of infection.

Additional data were extracted from medical records, including patient age, sex, type and date of primary surgery, date of revision surgery, cultures results, the presence of a sinus tract prior to surgery, or pus intraoperatively, both considered confirmatory criteria. Preoperative laboratory values including white blood cell (WBC) count, C-reactive protein (CRP) and erythrocyte sedimentation rate (ESR), obtained within five days from revision surgery, were also recorded. Clinical signs such as redness, swelling, pain – listed as suggestive criteria in Table 1 – were infrequently documented in the medical records and, when absent, rarely explicitly negated. A confirmed infection was defined as ≥2 positive cultures of the same bacterium, along with at least one of the confirmatory criteria listed in Table 1.

The preoperative stated reason for revision – as described in the records – was assessed using a three-point Likert scale ranging from 0 to 2. In a score of zero, there was no documented suspicion of infection; a score of one reflected vague wording or a low level of clinical suspicion and a score of two denoted a clear and explicit expression of a high likelihood of a deep SSI. An unexpected positive culture (UPC), was defined as a score of zero on the Likert scale accompanied by normal laboratory values and no additional confirmatory criteria – that is, no preoperative or perioperative indication of infection.

**Figure 1.**
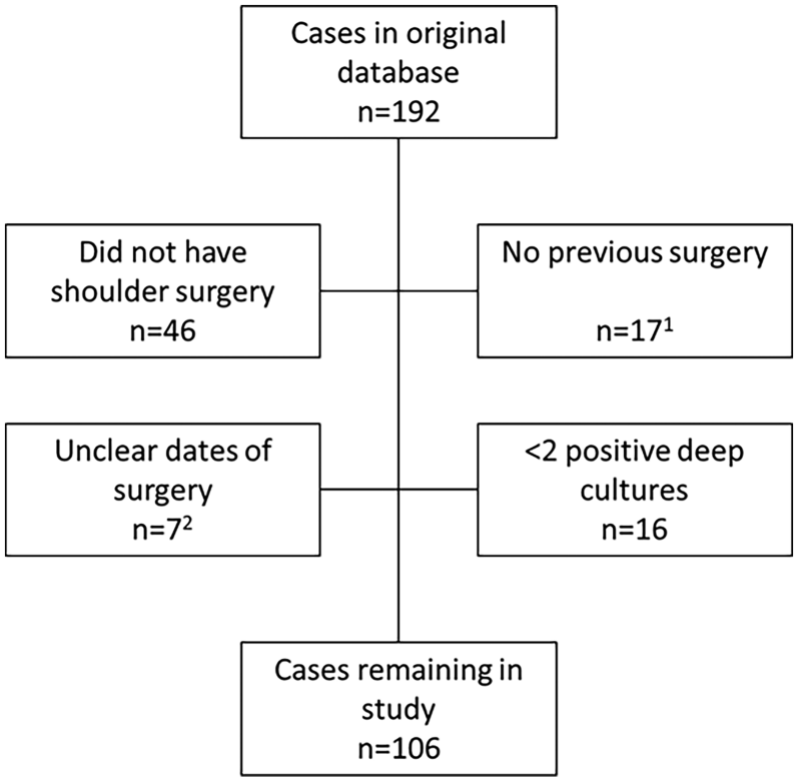
Study flowchart. (1) Among these: SAPHO 4 cases, septic arthritis 5 cases. (2) All patients with unclear surgery dates pertained to primary surgery. All these patients had primary surgery at another hospital, median ∼7 years prior.

Primary surgery was defined as the initial procedure performed on the shoulder that then subsequently was revised and cultured. All revision surgeries were carried out at the same institution. The database initially contained 192 patients who had undergone upper extremity surgery, of whom 106 patients remained for final analysis (Figure 1). With the exception of two acromioplasties, all primary procedures were performed as open surgeries.

### Sampling and culturing

For each case, five deep tissue cultures were collected using a standardised protocol, with each sample obtained using a separate sterile clamp or rongeur.^16^ In cases involving implants, tissue samples were taken immediately adjacent to metal/polyethylene, and, where applicable, from both sides of the joint. In cases of arthroscopic revision (2/106), five separate sterile instruments such as graspers, or meniscal punches were inserted through an arthroscopic cannula to minimise skin contamination.

All tissue samples were immediately put in thioglycolate broth. Cultures were maintained for seven days, with daily assessments for bacterial growth. In cases of visible broth growth of bacterium, suspected growth, or no apparent growth within seven days, the broths were further cultured on four different agar plates designed to enhance growth of various bacterial species: an anaerobic blood agar plate, a haematin blood agar plate, a chromogenic agar plate for Staphylococci, and a urinary tract infection agar plate. The plates were incubated at 35 °C under both aerobic and aerobic conditions, for two and four days, respectively. Finally, emerging bacterial colonies were identified with matrix-assisted laser desorption/ionisation time-of-flight mass spectrometry to categorise bacterial species.

Additionally, data from a separate local database were used to determine the total number of primary acute and elective procedures – including shoulder arthroplasty, clavicle and scapular surgery and AC-joint reconstructions – performed at our institution during the same period, January 2015 to December 2021. This enabled for a rough estimation of infection rates.

### Statistical methods

For comparing proportions, a Chi-square test with Yates correction was used. Binary logistic regression was used to assess the correlation between confirmed infection and laboratory values before revision surgery. Data distribution is expressed as median and range.

## Results

### Bacterial flora

Of the 106 cases, 15% (16/106) had undergone open rotator cuff repair, 25% (27/106) shoulder arthroplasty, 38% (40/106) had open reduction with internal fixation (ORIF). Among the ORIF cases, 29 involved proximal humerus fractures treated with either nailing or plating, 10 clavicle fractures managed with plates and two were scapular fractures. The remaining 22% (23/106) included arthroscopic acromioplasties, early and delayed treatment of AC-joint dislocations and a single case of glenohumeral arthrodesis.

Among the cases with at least two positive cultures for the same bacterium, *C acnes* was the most frequently identified pathogen, detected in 62% (66/106) of cases. In 49% (52/106), *C acnes* was the sole organism isolated. The next most common bacteria were *Staphylococcus epidermidis* (26/106) and *Staphylococcus aureus* (9/106), as detailed in Table 2. A total of twenty-one cases (21/106) had ≥2 positive cultures for two or more bacterial species. *C acnes* was the most prevalent pathogen in both men (41/76) and women (19/30).

**Table 2.** Distribution of 106 cases with ≥2 positive cultures for each species.

Containing *Cutibacterium acnes* (*n* = 66)		No *C acnes*^b^ (*n* = 40)
*C acnes* only (*n* = 52)	*C acnes* and other species (n = 14)	
	* Staphylococcus epidermidis* (n = 9)	*S epidermidis* (*n* = 17)
	*Staphylococcus aureus* (n = 0)	*S aureus* (*n* = 9)
	Other^a^ (n = 5)	Other^a^ (*n* = 15)

^a^ *Staphylococcus caprae/capitis, Enterococcus faecalis, Cutibacerium avidum, Carnobacterium divergenus, Escherichia coli, Klebsiella aerogenes.*

^b^ Some cases are polymicrobial, hence some bacteria are present in more than one case.

**Figure 2.**
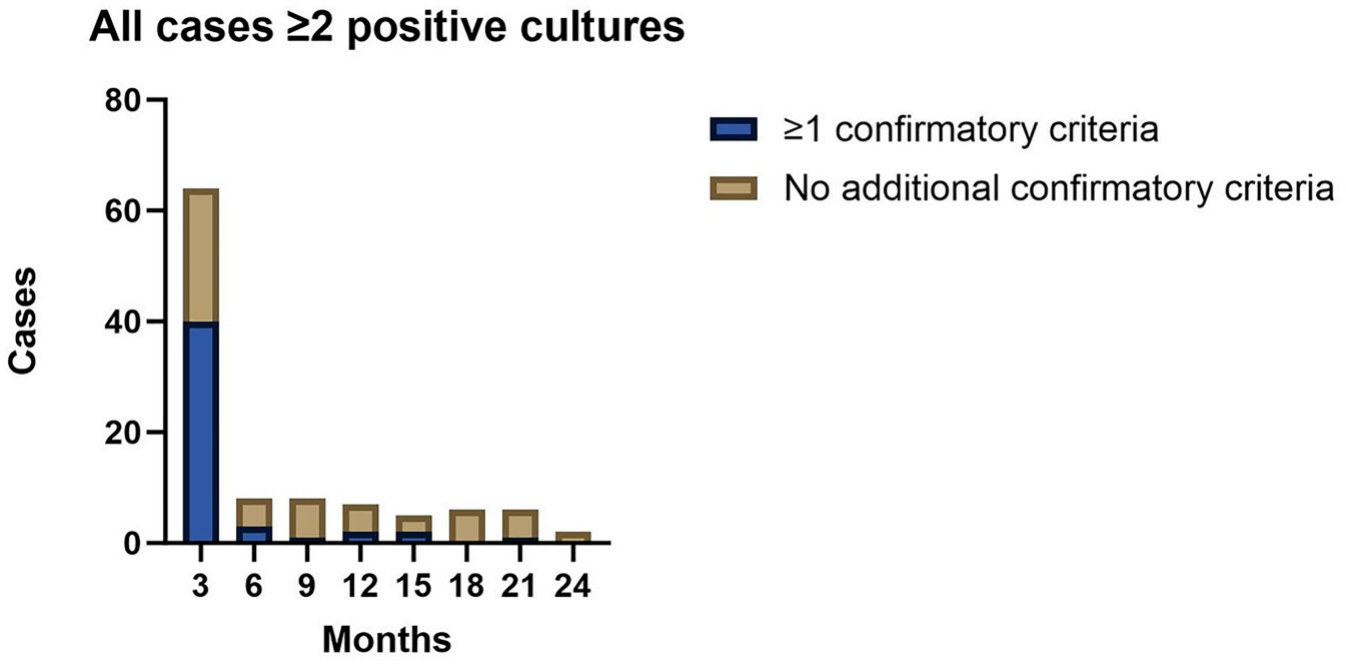
All cases with and without additional confirmatory criteria (*n* = 106).

The proportion of cases exhibiting additional confirmatory criteria as documented in the medical records is illustrated Figure 2 and Table 3. In total, 50 cases fulfilled this “double criterion”, of which 82% (41/50) had undergone revision <3 months of primary surgery*.* Twenty-five cases (24%) were classified as UPCs, of which seven occurred in revision arthroplasty. All UPCs were culture positive for *C acnes*; four of these were polymicrobial, each of them with a coagulase negative staphylococcus identified as the secondary organism.

**Table 3.** Bacterial growth in cases with both ≥2 positive cultures for at least one species (sp.) and one or more additional confirmatory criteria.

<3 months			3–24 months	
*Cutibacterium acnes* only	*C acnes* + 1/5 for other spp^a^	≥2/5 for more than one spp^b^	*C acnes* only	≥2 spp^c^
13/41	18/41	10/41	3/9	6/9

^a^ * Staphylococcus aureus* 1 case. Different CNS spp. 15 cases. Remaining *B. Cereus, C. avidum* 1 case each.

^b^ Different CNS spp. 8 cases. *C acnes* 3 cases. *F magna, B cereus* 1 case each.

^c^ *S aureus* 4 cases. Different CNS spp. 5 cases. Enterococci, *P aeruginosa*.

Neither leucocyte count nor CRP were predictive of a confirmed deep infection in the 60 cases where *C acnes* was the sole identified species. When included in a logistic regression model the OR was 0.878 (95%CI: 0.61–1.27, *p* = 0.49) for leucocyte count and 1.00 (95%CI: 0.98–1.01, *p* = 0.66) for CRP, respectively. See also Tables 4 and 5. When examining the relationship between the number of surgeries to gender, men had an overall higher risk of a positive culture (*p* < 0.001), and specifically of being positive for *C acnes* (*p* = 0.012).

**Table 4.** Patient demographic and lab values for cases undergoing revision surgery <3 months, 64 cases, laboratory values <5 days prior to surgery.

	All	*Cutibacterium acnes* (only)	Other bacteria
Age, median (range)	59 (23–87)	56 (23–87)	62 (26–82)
Sex, men, *n* (%)	50/64 (78%)	19/25 (76%)	31/39 (79%)
Leukocyte count [x10^9^/L], median (range)	8.4 (4.5–20.3)	8.6 (4.5–11.6)	8.1 (4.9–20.3)
CRP [mg/L], median (range)	39 (<5–204)	39 (<5–177)	52 (<5–204)
ESR [mm], median (range)	54 (6–98)	61 (7–98)	50 (6–97)

CRP: C-reactive protein; ESR: erythrocyte sedimentation rate.

**Table 5.** Patient demographic and lab values for cases undergoing revision surgery 3–24 months, 42 cases, laboratory values <5 days prior to surgery.

	All	*Cutibacterium acnes* (only)	Other bacteria
Age, median (range)	53 (18–77)	52 (18–77)	56 (19–74)
Sex, men, *n* (%)	27/42 (64%)	22/35 (63%)	4/7 (57%)
Leukocyte count (x10^9^/L), median (range)	7.6 (4.7–12.8)	7.5 (4.7–10.5)	8.3 (6.6–12.8)
CRP (mg/L), median (range)	28 (<5–92)	20 (<5–37)	61 (<5–92)
ESR (mm), median (range)	20 (10–82)	11 (10–27)	45 (12–82)

CRP: C-reactive protein; ESR: erythrocyte sedimentation rate.

### Cases revised within 3 months

Sixty-five of the 106 cases underwent revision surgery within the first 3 months following the primary procedure (Figure 2). In thirteen of these cases (20%) there was no documented suspicion of infection in the medical records corresponding to a score of zero on the Likert scale. These cases primarily involved early prosthetic revision surgery due to instability, fixation failure or a lack of preoperative documentation. In remaining cases, some degree of clinical suspicion was noted, typically related to wound dehiscence or a combination of unexpected pain and persistently abnormal laboratory values.

Among the confirmed infections in these group – 41 cases – *C acnes* was the sole identifiable organism in 13 of them (32%). In the remaining cases, at least one additional species was detected in one or more samples (Table 3).

### Cases revised 3–24 months postoperatively

**Figure 3.**
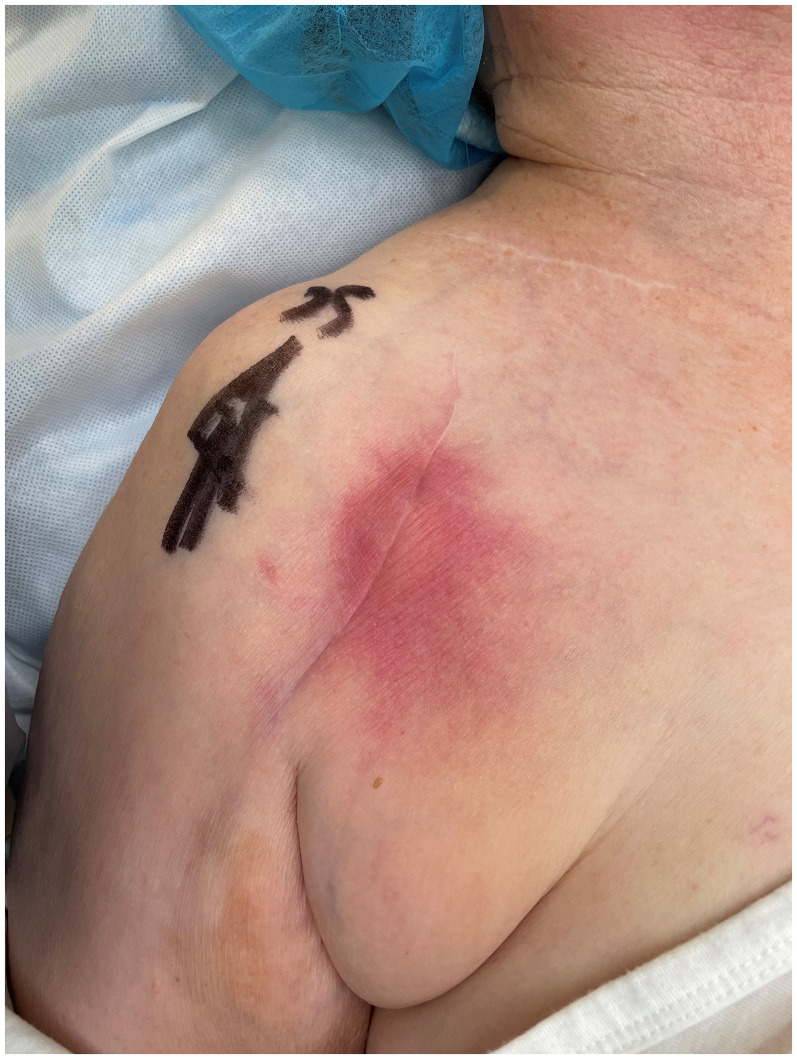
One patient presenting 4.5 months postoperatively after a reverse shoulder arthroplasty with a rapid onset of pain, followed within two weeks by redness and swelling. Intraoperatively, pus was found, and cultures revealed growth only of *Cutibacterium acnes* in 3/5 samples.

Among the 41 cases revised between 3 and 24 months postoperatively, 35 (85%) had ≥2 positive cultures for *C acnes*, with 33 of these showing *C acnes* as the only identified organism. Some degree of clinical suspicion was documented in eighteen cases, with the highest level of suspicion noted in 10 of the 41. Additional confirmatory criteria were observed in 9/41 of cases. In three of these, *C acnes* appeared to be the only identifiable pathogen, with one case that presented as late as 15 months after surgery exhibiting both a sinus tract and intraoperative pus (Figures 2 and 3). Among the later-diagnosed cases, three demonstrated growth of *S aureus*; notably one of them emerged 21 months after the index procedure, with a rapid symptom onset and growth of *S aureus* in 4/5 cultures.

### Incidence of infection

To estimate infection rates, only confirmed cases were included in the incidence calculations. Based on this, the estimated infection rates were as follows: 2.7% (9/330) for arthroplasties, 6.4% (15/235) for ORIF of the proximal humerus, 3.3% (10/301) for rotator open cuff repairs, 5.6% (4/72) for clavicle fractures and 8.3% (2/24) for ac-joint ligament reconstructions.

All patients meeting the confirmatory criteria were managed in accordance with established protocol, beginning with initial empirical intravenous broad-spectrum antibiotic therapy, followed by targeted treatment directed at the organism(s) deemed clinically significant. Where appropriate, drugs with biofilm activity were employed. While the subsequent clinical course varied between patients, all were ultimately assessed as free of infection. However, functional outcome remained highly variable.

## Discussion

This study provides strong evidence that *C acnes* is a clinically significant pathogen in shoulder infections, capable of eliciting an inflammatory response severe enough to produce purulence. In our cohort, *C acnes* was the sole identifiable bacterial organism in approximately 30% of the cases presenting with a sinus tract and/or pus. Furthermore, given that the presence of only one positive culture out of five of a species – according to prevailing consensus definitions^14,17^ – is considered indicative of a contamination, then *C acnes* was the only significant organism identified in two-thirds (34/50) of the confirmed cases.

Over the years, there has been a marked increase in the reported incidence of *C acnes* infections in the shoulder,^18^ likely attributable to the implementation of new protocols, including longer culturing durations. These improvements enhance the detection sensitivity of slow-growing bacteria but also introduce the risk of secondary contamination.^19^ This prompts questions regarding the clinical relevance of positive *C acnes* cultures.

In alignment with previous studies, we found *C acnes* to be the most frequently identified organism. In numerous instances, it was to be the only discernible pathogen when an infection was deemed likely. It is a slow-growing pathogen as illustrated by one case where pus was observed 15 months after primary surgery (similarly depicted in Figure 3). While *C acnes* frequently appears in polymicrobial infections, it remained the predominant single identifiable organism in 16/50 (32%) confirmed infections (see also Table 3).

UPCs are commonly encountered when routine culturing is performed in shoulder revision surgery, with *C acnes* representing the most prevalent isolate,^1^ as observed in our study. The clinical significance of such findings remains subject of ongoing debate and may not necessarily warrant treatment. Nevertheless, what constitutes an “unexpected” finding may vary between surgeons, and thus the interpretation of such results remains unsolved.

The estimated infection rates observed in this study were higher than those reported in previous literature (0.7–7%).^20–24^ It is conceivable that the true infection rate may even have been underestimated, as our calculations were based only on the cases with the highest probability of a bacterial infection. It is likely that a deep SSI originates from persistent skin bacteria from the patients themselves when standard recommendations regarding clean surgery are properly followed.^25^ From this perspective differences in infection frequency may be attributed to differences in surgical technique, duration of surgery,^26^ host factors such as co-morbidities and smoking^27^ or merely differing standards for defining and reporting infection. Comorbidities are likely to influence infection rates,^27^ but this aspect was beyond the scope of this study. It has consistently been shown that men have a higher rate of *C acnes* colonisation in the shoulder region.^28,29^ In our study, men were more likely to develop an infection following shoulder surgery and to be culture positive for *C acnes* in those cases.

Notably, there are a small number of cases exhibited positive confirmatory criteria beyond 3 months postoperatively (Figure 3), with the latest occurring 21 months postoperatively, following a rotator cuff repair. This patient presented with both a sinus and pus during surgery, with four positive cultures for *S aureus* and WBC, CRP and ESR all moderately elevated (10.6 × 10^9^/L, 61 mg/L and 12 mm, respectively). We hypothesise that this case may represent an instance of late haematogenous spread, whereas the others reflect the presence of slow-growing pathogens as well as the complexity of deciding on surgical revision under such circumstances.

This study is subject to certain limitations inherent to its retrospective design. To be included, patients had to undergo revision surgery where deep cultures were obtained. This decision is made by either a single or a small group of surgeons, and the rationale is always multifactorial – often difficult to fully determine in retrospect. For the same reason, information regarding wound drainage, sinus or pus during surgery was not always clearly recorded in the medical files, potentially leading to some “confirmatory cases” being overlooked in the analysis. The conclusion that *C acnes* is the sole causative pathogen is based on the assumption that the entire culturing process – from sampling to incubation – at our institution is reliable and valid. Additionally, we also assume that sinus/pus after surgery is the result of an infectious process, though, in theory alternate causes cannot be entirely ruled out.

A key limitation of this study – one shared by numerous other retrospective studies on SSIs – is the lack of detailed information in medical records regarding the decision-making process. Whether defining infection by an ICD code in a registry or extracting it from medical records, there might both be cases missing or, conversely, overestimated. Consequently, caution must be exercised when making comparisons across different studies or between various time periods before and after interventions,^30^ as this can be ambiguous.

## Conclusion

*C acnes* appears to be a clinically significant pathogen rather than a mere ‘red herring’^2,5^ or a contaminant during the culturing process.^12^ Nevertheless, it may occasionally be present without causing an active infection – more frequently so than other bacterial species.^1^ Given the frequent finding of *C acnes* in postoperative infections of the shoulder, which is also applicable to thoracic and spine surgery, it seems essential to adopt targeted strategies to combat this bacterium. At present, we believe this should include appropriately timed antibiotic prophylaxis,^31,32^ with coverage against *C acnes*^30,33^ as well as preoperative topical treatment with benzoyl peroxide.^29^
